# Resolving Male Infertility: A Case Report on Treating Obstructive Azoospermia Using SpermMobil in Intracytoplasmic Sperm Injection Procedure

**DOI:** 10.7759/cureus.55323

**Published:** 2024-03-01

**Authors:** Pavan Tej, Charu Pareek, Avanti Kalbande, Pranita A Bawaskar, Ankit Badge, Nancy Nair

**Affiliations:** 1 Clinical Embryology, School of Allied Health Sciences, Datta Meghe Institute of Higher Education and Research, Wardha, IND; 2 Obstetrics and Gynaecology, School of Allied Health Sciences, Datta Meghe Institute of Higher Education and Research, Wardha, IND; 3 Microbiology, Datta Meghe Medical College, Datta Meghe Institute of Higher Education and Research, Wardha, IND

**Keywords:** intracytoplasmic sperm injection, microscopic epididymal sperm aspiration, spermmobil, male infertility, obstructive azoospermia

## Abstract

Infertility affects couples worldwide. Among these, obstructive azoospermia (OA) is a common cause. In some cases, the lack of spermatozoa in ejaculation results from blockages in the male reproductive tract. In this case study, we discuss an infertile male diagnosed with OA following three years of unsuccessful attempts at conception. The male had a history of bilateral inguinal hernia repair due to congenital bilateral absence of the vas deferens. Diagnostic assessments confirmed azoospermia. Microscopic epididymal sperm aspiration (MESA) was performed for sperm retrieval due to its efficacy and reduced postoperative pain, testicular atrophy, and decreased testosterone levels. The retrieved sperm was processed using SpermMobil media for intracytoplasmic sperm injection. Following successful fertilization, embryo transfers resulted in a positive pregnancy test. This case highlights the significance of specific treatment approaches for OA, specifically the effectiveness of MESA and SpermMobil in achieving successful outcomes in assisted reproduction technology for male infertility.

## Introduction

Infertility is the inability to conceive after 12 months of unprotected regular sexual intercourse [1]. Infertility has become a severe medical issue that affects 8-12% of couples globally [2]. Male factors causing infertility, such as testicular dysfunction, endocrine disorders, factors associated with lifestyle, including tobacco and obesity, congenital anatomical factors, exposure to gonadotoxic agents, and aging, are all possible causes of infertility or decreased fertility [3]. Male infertility is evaluated with a thorough medical history, physical examination, and specific laboratory investigations, such as semen analysis, fructose test, and hormonal profile. Obstruction within the male reproductive tract can lead to infertility, resulting in the abnormal expulsion of semen. Inguinal hernia affects 4%-5% of male partners in infertility cases. Mesh is utilized for approximately 80%-90% of hernia repairs today [4]. Azoospermia can be described as the absence of sperm in semen. Diagnosis typically involves centrifuging a semen specimen for 15 minutes. Furthermore, confirmation requires examining at least two semen samples obtained more than two weeks apart [5]. The prevalence of azoospermia is around 10%-15% among infertile males [6]. In 60% of cases, non-obstructive azoospermia (NOA) or obstructive azoospermia (OA) are the primary causes of infertility [7]. In OA, there are high chances of sperm production, but an obstruction inhibits the spermatozoa from entering the ejaculate. On the other hand, NOA is a complex condition where testicular failure leads to no sperm production [8]. Testicular sperm extraction (TESE) is the most popular sperm extraction procedure for OA because it requires no surgical skill. However, TESE may cause postoperative pain as well as testicular atrophy and decreased testosterone levels [9]. Microscopic epididymal sperm aspiration (MESA)-intracytoplasmic sperm injection (ICSI) has better fertilization, clinical pregnancy, and delivery rates [10]. Moreover, the patient’s postoperative pain is reduced, the number of sperm collected is increased, the pressure on the embryologist handling the obtained sperm is reduced, and ICSI can be performed more efficiently. MESA, instead of TESE, should be used for OA patients [9]. In patients with inactive spermatozoa, SpermMobil is generally used to find highly active sperm for ICSI. SpermMobil is a HEPES-buffered reagent containing low bicarbonate and is utilized in clinical settings to perform in vitro examination of immotile sperm cells (immotile) extracted from testicular tissue because it provides motility to immotile sperm cells by activating cyclic adenosine monophosphate-dependent pathways [11].

## Case presentation

A 29-year-old man and a 26-year-old woman came to an infertility center in Maharashtra, India, after three years of marriage. They had been trying to conceive for the past three years without using any contraception. The primary concern of the couple was seeking infertility treatment, and they denied donor sperm or oocytes. There were no particular symptoms regarding infertility in the woman. They received a description of all treatments, benefits, and drawbacks, and informed consent was obtained.

Clinical history

The couple had a history of a failed in vitro fertilization (IVF) cycle in 2022. The female partner had regular menstruation cycles of ±28 days and no documented problems with conception, indicating that the problem was probably associated with the male partner’s fertility. The male had a history of bilateral inguinal hernia repair over five years. He was diagnosed with congenital bilateral absence of the vas deferens (CBAVD) by a urologist, and the transrectal ultrasonography (TRUS) report showed the absence of a vas deferens. Urologists also recommended sperm cell retrieval techniques. They did not have any sexually transmitted diseases. There was no significant history of inherited conditions in immediate family members. The male patient consumed tobacco products and drank alcohol occasionally. The female partner led a healthy life.

Clinical findings

The couple’s overall physical health was normal. The patient’s vitals were within the normal range. The male patient had typical secondary sexual characteristics, which indicated testosterone production. On external genitalia examination, the testicles and scrotum appeared normal. On physical examination, surgical scars from previous hernia repairs were found, and palpable masses were absent in the scrotum. This could indicate a recurrence of the hernia or complications from previous surgery. The patient’s semen analysis revealed a sperm concentration of 0/mL in 1.4 mL of semen. Multiple samples of semen from the patient were found to be free of spermatozoa. The fructose test was recommended to the patient to verify azoospermia. The test gave a negative result following several fructose tests, indicating the presence of fructose in the ejaculate. The results obtained from semen analysis, as shown in Table 1, suggested that the male patient was suffering from azoospermia.

**Table 1:** Results of the male semen analysis.

Semen parameters	Results	Reference value
Ejaculatory abstinence	7 days	2–7 days
Volume	1.4 mL	1.4–5mL
Appearance	Gray opalescent	Gray opalescent
pH	7.5	7.2–7.8
Sperm count	0 million/mL	≥16 million
Total sperm motility	Nil	40–43%
Morphology	Nil	3.9–4.0%

The hormonal profile of the male patient results revealed that the anti-Mullerian hormone level was 3.7 ng/mL, falling within the reference range of 1.0-4.0 ng/mL. Follicle-stimulating hormone level was 15 mIU/mL, exceeding the reference range of 3.5-12.5 mIU/mL. Meanwhile, the luteinizing hormone level was 13 mIU/mL, within the reference range of 2-15 mIU/mL. The testosterone level was 7.2 mIU/mL, slightly above the reference range of 0.8-7.6 mIU/mL. Estradiol level was 40 pg/mL, within the reference range of 20-55 pg/mL. The hormonal profile is shown in Table 2.

**Table 2:** Hormonal profile of the male patient. FSH = follicle-stimulating hormone; LH = luteinizing hormone; AMH = anti-Mullerian hormone

Hormone	Findings	Reference range
Serum AMH	3.7 ng/mL	1.0–4.0 ng/mL
FSH	15 mIU/mL	3.5–12.5 mIU/mL
LH	13mIU/mL	2–15 mIU/mL
Testosterone	7.2 mIU/mL	0.8–7.6 mIU/mL
Estradiol	40 pg/mL	20–55 pg/mL
Inhibin B	120 pg/mL	125–215 pg/mL

Diagnosis

The male patient was diagnosed with CBAVD based on the results of the semen-related examination and TRUS, which showed the absence of a vas deferens.

Therapeutic intervention

**Figure 1:**
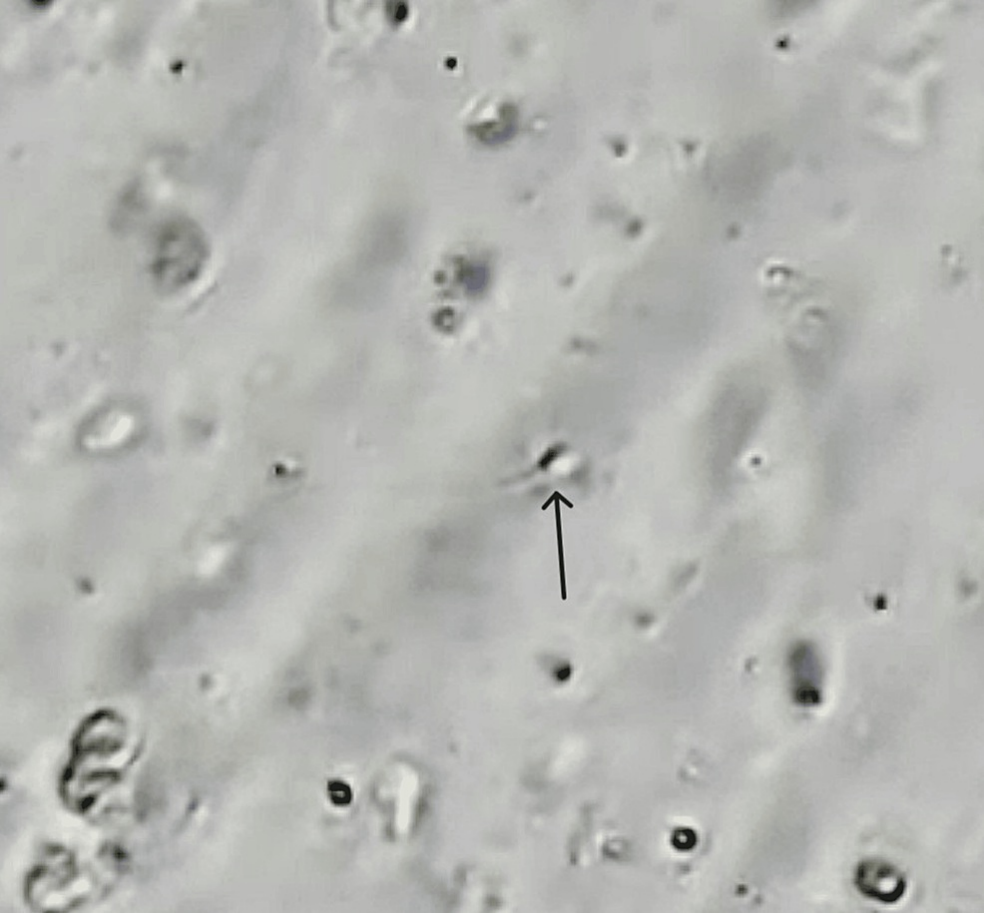
Spermatozoa obtained from microepididymal sperm aspiration. The arrow points to the spermatozoa.

In 2023, the patient first visited the Wardha Test Tube Baby Centre (WTTBC). The couple underwent counseling before the procedure. The female patient’s oocyte pickup (OPU) preparation began with a quick antagonist procedure. Before administering the gonadotropin-releasing hormone antagonist, a dominant follicle was induced with follicle-stimulating hormone or human menopausal gonadotropin until it reached a size of 14-17 mm. TESE is one of the most popular sperm retrieval techniques for azoospermia, including OA, because it requires no surgical skill. However, TESE may cause some complications, such as postoperative pain, as well as testicular atrophy and reduced testosterone levels. Figure 1 shows extracted sperm using the MESA technique; non-motile spermatozoa were occasionally recovered. The micropuncture technique can be used to obtain a large quantity of uncontaminated sperm for ICSI using MESA [11]. MESA typically yields more sperm than TESE, allowing for excess cells to be stored for future ICSI cycles. TESE should only be performed for men with OA when virtually no epididymal spermatozoa are present [12]. Sperm wash media (1 mL) was added to the fluid containing sperm (1.2 mL). The sample was centrifuged at 800 rpm for 8-10 minutes, the supernatant was discarded, and the pellet was saved for future use. The sperm pellet was re-suspended in 0.3 mL of SpermMobil media, and the sample was incubated at 37°C for 30 minutes. The prepared sample was used for ICSI.

**Figure 2:**
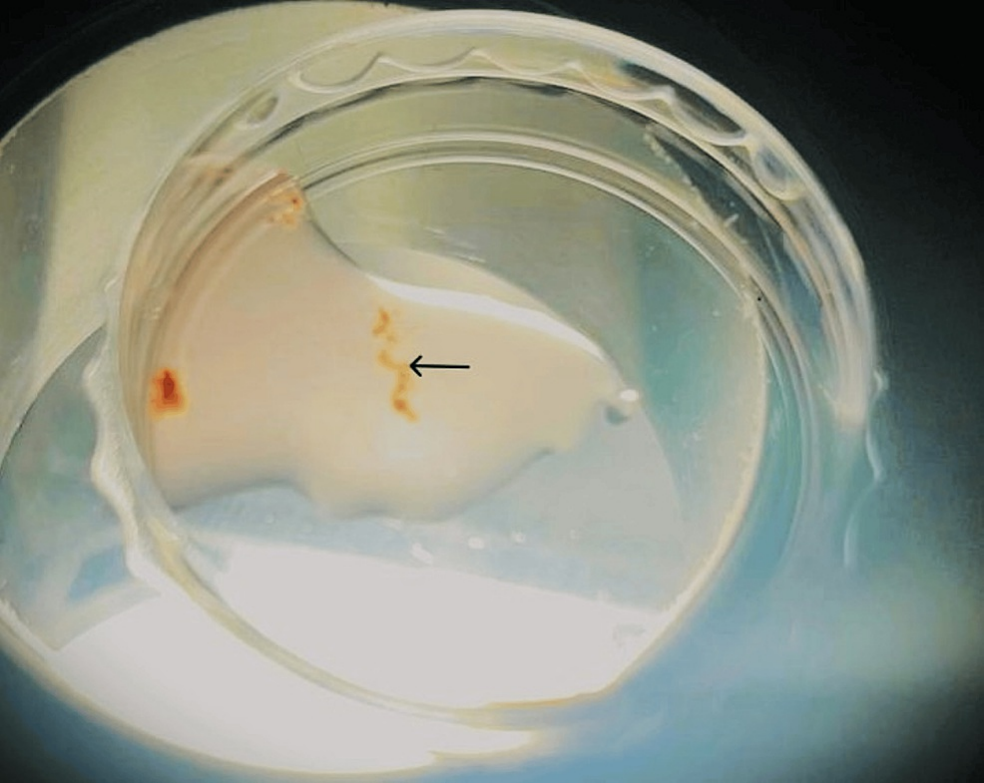
Seminiferous tubule retrieved by microepididymal sperm aspiration. The arrow points to the seminiferous tubule.

MESA was performed using a micropuncture procedure under the influence of local anesthesia and a spermatic block. Once spermatozoa were retrieved from testicular tissue, sperm cells were separated from the surrounding cells, as shown in Figure 2.

Follow-up

A total of 10 oocytes were retrieved after OPU, seven of which were in the M2 stage of metaphase. One oocyte was in the M1 stage of metaphase, and one oocyte was in the germinal vesicle phase. ICSI was performed on seven mature oocytes. On day one of observation, five oocytes were fertilized. On day five of the observation, one blastocyst was formed. A frozen embryo transfer was done after two menstruations on day 12 of the female patient’s menstrual cycle. The urine pregnancy test results were positive, and serum beta-human chorionic gonadotropin showed a pregnancy with a value of 1,132 mIU/mL. Ultrasound indicated the development of one single sac during gestation.

## Discussion

This case study demonstrates the successful pregnancy outcome in an infertile couple using SpermMobil media and sperm retrieval surgery employing MESA for OA to retrieve an adequate number of motile sperm with minimal damage to the patient [10]. The use of SpermMobil in samples containing mostly immotile sperm not only makes the embryologist’s job easier but also improves treatment outcomes for patients with poor prognoses, as reported in a case report on obstetric and perinatal outcomes after using a theophylline derivative in human clinical trials [13]. Verheyen et al. [14] explained that SpermMobil is a commercially available product. To use it, a 10 μL droplet suspension is first smeared on the bottom of a dish and overlaid with oil. Then, 3 μL of SpermMobil is added to the dish. The activating effect of SpermMobil starts within 10 minutes and lasts for a maximum of one hour. Before being injected, motile sperm are collected and washed in sperm buffer [14]. SpermMobil is specifically designed to enhance sperm movement. In cases of OA, where sperm motility may be compromised, SpermMobil may provide additional benefits, such as improving sperm quality and increasing the success of ICSI. Pareek et al. [8] concluded that NOA is effectively managed by combining theophylline, pentoxifylline, and hyaluronic acid. The findings indicate the efficacy of these methods in reactivating sperm production, providing an innovative approach to treating male infertility. Theophylline, one of the significant components of SpermMobil, was used in the management of OA and obtained a successful rate in ICSI. Carrageta et al. [11] discussed that the Genipin-treated spermatozoa were washed twice in phosphate-buffered saline before being incubated in Biggers, Whitter, and Whittingham medium supplemented with SpermMobil medium (1:10 dilution, v:v) for 20 minutes at 37°C. Following every procedure, overall and progressive motility were assessed [11]. In our case, SpermMobil enhanced the sperm’s ability to fertilize oocytes because it improves the progressive motility of sperm, reduces oxidative stress, and increases the capacitation process. This case highlights the significance of individualized fertility treatments customized to a couple’s specific needs, allowing them to fulfill their desire for parenthood. The efficiency of surgical procedures followed by ART intervention can vary depending on individual patient factors such as the nature and extent of the obstruction and the quality of the retrieved sperm [15].

## Conclusions

A successful pregnancy was achieved through a combination of MESA followed by ICSI using SpermMobil. MESA was ultimately performed due to its effectiveness and reduced postoperative complications. Moreover, the success of this case demonstrates the progress made in reproductive medicine and the efficacy of advanced ART. ICSI using SpermMobil can be a good choice for promoting embryonic growth and improving pregnancy rates.
